# Research on Heat Dissipation of Multi-Chip LED Filament Package

**DOI:** 10.3390/mi13010077

**Published:** 2021-12-31

**Authors:** Lipeng Tan, Peisheng Liu, Chenhui She, Pengpeng Xu, Lei Yan, Hui Quan

**Affiliations:** 1 Jiangsu Key Laboratory of ASIC Design, College of Information Science and Technology, Nantong University, Nantong 226019, China; tanlipengdsg@163.com (L.T.); 1811310001@stmail.ntu.edu.cn (C.S.); 2110320054@stmail.ntu.edu.cn (P.X.); 2110310042@stmail.ntu.edu.cn (L.Y.); 2 College of Science, Nantong University, Nantong 226019, China; 2102310022@stmail.ntu.edu.cn

**Keywords:** LED, finite element analysis, heat dissipation, illuminance, illuminance uniformity

## Abstract

By studying the substrate material, structure, chip distribution, and array form of the multi-chip light-emitting diode (LED) package, the heat-dissipation capacity of the LED package is improved. Finite element analysis and steady-state thermal analysis are used to simulate and analyze LED packages with different materials and structures. Using the theory of LED illuminance and uniformity, the illuminance of some structures is computed. The results show that the change of substrate material and structure can greatly impact heat dissipation, while changing array forms has little effect on heat dissipation. By improving the spatial distribution of the chip, the temperature superposition problem of the substrate is solved, and the illuminance and uniformity are improved while dissipating heat. The LED filaments of the combined, equidistant, chip-distribution mode have improved heat dissipation. The S-type equal difference has the highest illumination and high illumination uniformity.

## 1. Introduction

A light-emitting diode (LED) is a solid-state semiconductor device that can directly convert electrical energy into visible light [1]. LEDs are fourth-generation illumination sources, which have many improvements—such as higher luminous efficiency, faster startup, longer life, higher reliability, better energy savings, and increased environmental friendliness [2]—that help them meet the needs of various applications, such as displays [3,4], decoration, backlight, and lighting [5,6]. Multi-chip packaged LED arrays were designed to make the light output consistent with or even exceed that of traditional light sources [7]. Compared to traditional LED light sources, LED filaments realize 360-degree full-circumference light output [8], which can reduce the number of optical devices needed and decrease optical loss [9], thereby achieving higher lumen output. However, LED luminous efficiency can only reach 10% to 20% of the input power currently, with the remaining 80% to 90% of energy converted into thermal energy [10]. Moreover, LED filament lamps require simple and stylish designs, prohibiting the implementation of bulky heat sinks such as those used in traditional patches or light sources of chip on board (COB). Therefore, the heat-dissipation problem of LED filaments is a major bottleneck for the widespread application of LED lights. The substrate material, size of heat dissipation holes, spatial distribution of the chips, and the array mode are studied in order to determine the influence of different methods for heat dissipation of the LED filament package. This study aims to lay the foundation for obtaining the best heat-dissipation performance and to guide future LED filament design.

## 2. Thermal Analysis of LED Filament Packaging

The temperature distribution of single-chip LED packages is related to the eccentricity of the chip. The larger the eccentricity of the chip, the higher the temperature of the substrate, with the central temperature lower than the temperature of the chips on both sides of the substrate [11]. Since each chip in a multi-chip LED filament has a rated heating power, the heat between each chip is superimposed after removing the influence of external temperature and humidity, which cause an uneven heat distribution on the module interface [12]. LED-chip heat dissipation is linked to the substrate and circuit selection process. Higher temperature causes the gold wires between the connected chips to break due to excessive internal stress, resulting in an open circuit and affecting the light-emitting quality of the LED [13]. As a result, it is imperative to solve the heat dissipation problem of multi-chip packaging.

**Figure 1:**
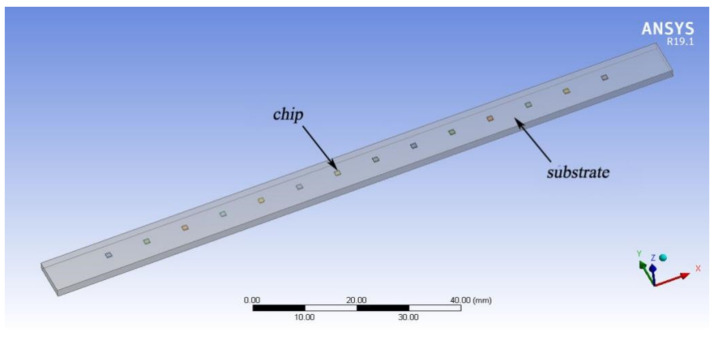
Model package of the LED filament.

The LED filament is comprised of a chip, a substrate, a gold wire, silica gel, etc., and the chip and the substrate are connected by a bonding material. The finite element calculation software is used to build a 3D model of an LED filament [10]. In order to more easily facilitate analysis, the solid crystal layer, electrode lead, silver-plating layer on the surface of the substrate, and the filling layer of the chip were omitted in the model; also, the chip is directly fixed without considering the contact thermal resistance of each layer. The LED chip size is 1 × 1 × 0.25 mm^3^; the thermal conductivity is 130 W·m^−1^·K^−1^; the substrate size is 129 × 8 × 2 mm^3^; and the thermal conductivity is 20 W·m^−1^·K^−1^. The simplified model of an LED filament built in the finite element analysis software consists of the substrate and LED chip. The number of chips on the substrate is 14. The model package of the LED filament is presented in Figure 1.

In the simulation of the LED filament, the air convection coefficient on all surfaces was set to 10 W/(m^2^·K), and the ambient temperature was set to 25 °C. Assuming that the input power of the chip is 93.75 W [14], and 80% of the chip power is converted into thermal energy, then the thermal power (P) is 75 W. As the applied load is equal to the quotient of the thermal power of the chip and the volume of the chip, the applied load of the model chip can be obtained at 3 × 10^8^ W/m^−3^. The highest temperature simulated by the LED filament is 68.388 °C, the lowest temperature is 60.818 °C, and the temperature difference is 7.57 °C. The middle temperature of the LED filament is higher than what is on both sides, which accelerates the failure of the middle chip of the LED wick. Therefore, it is necessary to study the influence of substrate and chip arrangement on the heat dissipation of the LED package in order to propose the best solution.

## 3. Research on Heat Dissipation of LED Filament Packaging

### 3.1. Influence of Substrate Material on LED Filament Packaging

The substrate fulfills two critical functions in the LED package: the LED lamp beads are connected to the upper surface, and the heat sink is installed on the lower surface. In other words, the package substrate plays the role of a bridge. Crucially, for high-power LED device packages, the substrate is the main channel for the diffusion of the LED chip’s heat into the environment. Thus, the optimal substrate material for adequate heat dissipation must have high electrical insulation performance, high stability, and high thermal conductivity, and its thermal expansion coefficient, flatness, and high strength must be consistent with the chip. The heat dissipation substrate material of LED filaments is generally Al_2_O_3_. The thermal conductivity of Al_2_O_3_ is 20 W·m^−1^·K^−1^. To study the influence of substrates with differing thermal conductivities on the heat dissipation of an LED filament, we selected four common non-metal substrates: Al_2_O_3_ [15], AlSiC [16], AlN [16], and Si [17], as well as two kinds of metal substrates: aluminum [17] and copper [17,18]. The thermal conductivity of the above-mentioned substrates is provided in Table 1.

**Figure 2:**
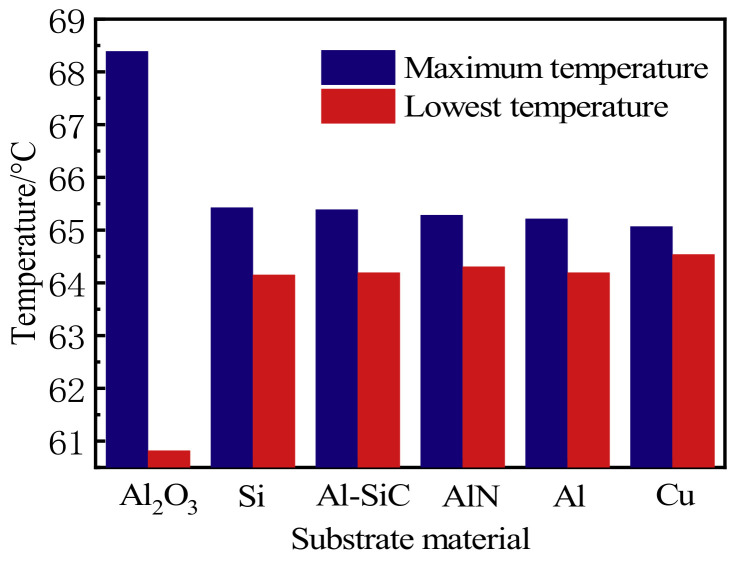
The maximum and minimum temperature of different substrate materials.

As shown in Figure 2, the high thermal conductivity of the substrate helps to reduce temperature rise. With the increase of thermal conductivity, the rate of change of high thermal conductivity is smaller than that of low thermal conductivity, which indicates that the gap of its heat dissipation effect will narrow when the substrate reaches a certain threshold of thermal conductivity. This is compatible with the conclusion of Yu. Yu et al. [19] proved that improving the thermal conductivity of the substrate has no observable effect on improving the performance of the device while the thermal conductivity of the substrate is greater than a specified value. Figure 2 shows the temperature difference between the highest temperature and the lowest temperature for different substrate materials. The higher the thermal conductivity, the smaller the temperature difference. Moreover, as the temperature difference decreases, the heat resistance of the substrate in the heat dissipation process will decrease the temperature transmission barrier, so that the uniformity of the LED package becomes better and better. In summary, the heat dissipation effect of the Si, AlSiC, AlN, Al, and Cu substrates is preferable to the original substrate, and the temperature difference of Cu is the smallest.

**Figure 3:**
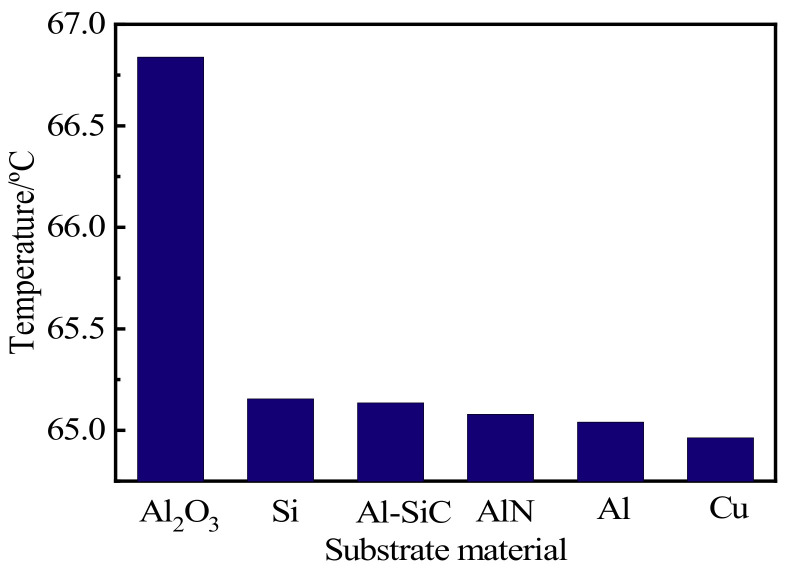
Junction temperature of different substrate materials.

The quality level of LED packages and modules, measured by performance indicators such as the amount of light, color temperature, and other optical properties, is closely associated with the junction temperature of the internal chip of the LEDs. The higher the junction temperature, the worse the LED performance will be [20]. When current is applied to the LED, the current will promote electron movement inside the PN junction, promote the compound release of energy between electrons and holes, and will also release heat. Most of the CSP-LEDs are integrated light sources. The temperature of the LED will not rise after being lit, and it will tend to be stable when it rises to a certain value in the normal environment. The stable temperature is the junction temperature. Therefore, the significance of junction temperature analysis equals that of heat dissipation analysis [21]. The heat dissipation effect can be expressed by the calculation of junction temperature. Since the LED filament is a multi-chip device, we are required to calculate its average junction temperature. Based on the finite element analysis of the chip temperature, the maximum junction temperature on each chip can be obtained. The average junction temperature of the LED filament can be calculated by dividing the sum of the junction temperature on all chips of the total number of chips. The junction temperature of six diverse substrate materials is displayed in Figure 3.

**Figure 4:**
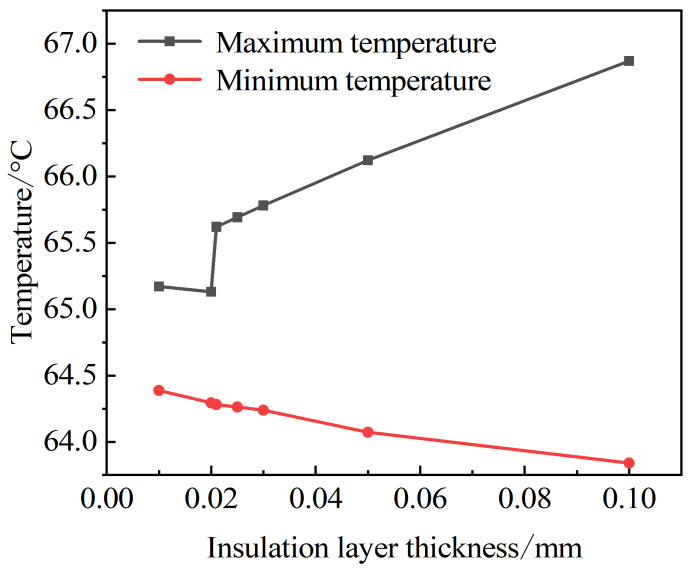
LED temperature under different insulation layer thicknesses.

All five materials proposed reduced the junction temperature, and the junction temperature of the metal material Cu is the lowest. However, for safety, metal substrates need an insulating material or to be coated with an insulating film to prevent a short circuit or leakage caused by contact between other conductors and the substrate [22]. Therefore, Figure 4 shows the metal Al as an example of the temperature change of a multi-chip LED with different insulation layer thicknesses.

It can be seen from the above figure that the difference between the highest and lowest temperatures is the smallest when the insulation layer is about 0.02 mm, which means that the best insulation thickness is about 0.02 mm. When the thickness is less than 0.02 mm, the maximum temperature decreases as the thickness increases, and when the thickness is greater than 0.02 mm, the maximum temperature increases as the thickness of the structure increases. Above 0.02 mm, the highest temperature suddenly increases due to the temperature change caused by the different interface-material properties.

Therefore, the thermal conductivity and thickness of the insulating layer also affect the heat dissipation of the LED package. The ceramic substrate itself is an insulating material and can be directly encapsulated on the substrate. Thus, compared to the other five materials, AlN has better heat dissipation performance among the non-metallic materials.

### 3.2. Influence of Substrate Structure on LED Filament Packaging

**Figure 5:**
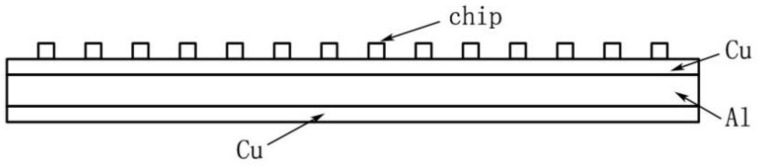
Schematic diagram of the composite substrate.

The choice of the packaging substrate and its structure is critical to the heat dissipation of the LED. The higher the thermal conductivity of the substrate material, the better the heat dissipation performance of the LED device, but it is necessary to appropriately select a substrate with high heat dissipation. However, Li [23] pointed out that when the thermal conductivity of the substrate is greater than 300 W/(m·K), the difference in thermal resistance generated by the substrate is not large, so there is no need to blindly pursue materials with high thermal conductivity. It can be seen from the previous section that the heat dissipation performance of metal substrates is better, has a cost advantage, and the process is relatively mature, so the temperature of the LED package can be reduced by using a combination of metal-composite substrate and insulating layer. The material of the substrate of the LED filament is mainly aluminum. Some businesses directly use substrates made of copper to improve the heat dissipation efficiency, but increased production costs make this a poor choice. Further, the thermal shrinkage of copper and aluminum is similar, and the composite substrate can be manufactured by the high-temperature explosive composite method and the sputtering method (first layer: copper; second layer: aluminum; third layer: copper) [24]. Figure 5 shows the model diagram of the LED filament package using the composite board.

Utilizing the finite element analysis to simulate the structure reveals that the highest temperature is 65.11 °C, the lowest temperature is 64.48 °C, the temperature difference is 0.63 °C, and the junction temperature is 64.98 °C. The result is similar to pure copper, but with a much lower cost. When using a composite substrate, certain impurities in the metal will increase its resistance. The influence of impurities on metal resistance depends on the type, content, and state of impurities in the metal. Therefore, the influence of resistance cannot be ignored in the simulation of Cu/Al/Cu composite substrates.

**Figure 6:**
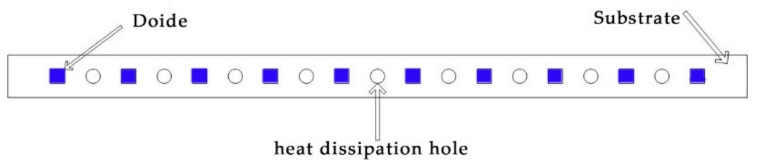
The model of a multi-chip LED package containing heat dissipation holes.

In addition to the above-mentioned method of modifying the structure of the substrate to promote heat dissipation, further improvement can be obtained by using heat dissipation holes. Xu et al. [24] proposed a ceramic substrate structure with copper-filled hot holes to improve the thermal management and lifetime of DUV-LEDs. Compared to the traditional structure, the thermal resistance of the DUV-LED with 4×4 thermal holes is reduced by 23.04%. Lin et al. [25] simulated and measured the area of the through-aluminum-nitride-via (TAV) substrate and the thermal stress of the Cu/AlN bi-material plate during thermal loading to make the TAV substrate with high thermal conductivity provide better heat dissipation. Additionally, many patents mention the utilization of heat holes to improve the heat dissipation performance of the filament. Therefore, it is feasible to arrange heat dissipation holes on the substrate. This section mainly studies the optimization of heat dissipation hole size. To facilitate the simulation, we assume that the heat dissipation hole is circular. The area of the heat dissipation hole is adjusted by changing its radius. The model of a multi-chip LED package containing heat dissipation holes is shown in the Figure 6. The radius of the heat dissipation hole is set to 0.5 mm, 0.75 mm, 1 mm, 1.25 mm, 1.5 mm, or 1.75 mm.

The heat convection formula is as follows:

(1) Q=hAΔT

When the air convection coefficient h and the fluid-solid temperature difference ΔT remain unchanged, increasing the radius of the radiating hole can increase the contact area A and increase the heat flow.

**Figure 7:**
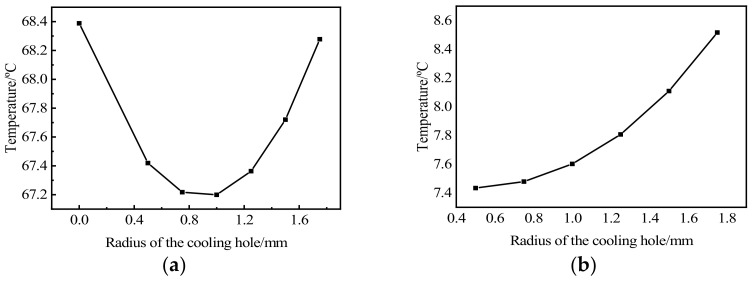
(**a**) Maximum temperature; (**b**) Temperature difference.

Figure 7a exhibits the maximum temperature corresponding to the heat dissipation holes of different areas. The heat dissipation is highest and the maximum temperature lowest with a heat dissipation hole radius of 1 mm. This is because the heat dissipation hole can increase the area of the air convection surface, resulting in more heat being released into the air and thus greater heat dissipation than the traditional structure. With the further increase of the radius, the influence of the change of substrate structure on the heat dissipation becomes larger, resulting in the increase of temperature. Figure 7b reveals the simulated temperature difference between the highest and lowest temperatures of different radii of heat dissipation holes. The increase of the heat dissipation aperture leads to a larger temperature difference, which indicates that the heat dissipation hole can achieve a beneficial effect of reducing the heat of the chip, but the lack of uniformity requires further research.

### 3.3. Impact of the Spatial Distribution of the Chip on LED Filament Packaging

Existing LED filaments are mostly arranged at equal intervals [26]. Chen et al. [27] optimized the thermal management scheme of COB based on glass substrates by studying the effects of substrate material, substrate thickness, chip gap, chip size, and convection conditions on COB heat dissipation. The optimized arrangement of the LED filaments can be used in actual production. It is proposed that the influence of the chip gap on the LED junction temperature is greater than that of the substrate thickness, therefore it is essential to design the chip distribution reasonably. He et al. [28] conducted thermal analysis on a COB array soldered onto a heat sink and found that increasing the chip gap and the thickness of the copper circuit layer reduces the junction temperature effectively. She et al. [14] designed three innovative LED filaments and conducted related experiments and simulations which showed the feasibility of reducing the junction temperature of the LED filament. Besides equal interval and arithmetic, two other LED packaging structures are studied in this research design: S-type arithmetic and combined equidistant LED. These arrangements effectively avoid the difficulty associated with central-chip heat dissipation due to small chip spacing. Moreover, this method not only improves LED heat dissipation, but also enhances the illuminance and uniformity of the LED filament.

**Figure 8:**
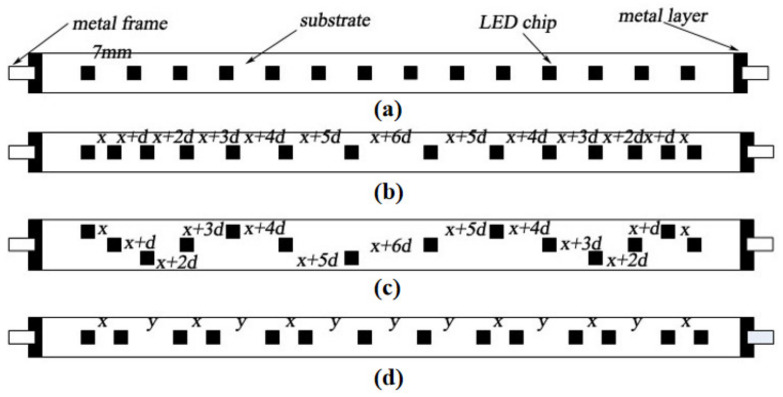
Model diagrams of packages with different chip distributions: (**a**) equidistant distribution; (**b**) arithmetic distribution; (**c**) S-type distribution; (**d**) combination-type distribution.

The pitch of the equally spaced chips is set to 7 mm. The arithmetic distribution of the chips arranges the chip pitch from the edge to the center in an arithmetic progression (x + nd) and distributes the chips symmetrically along the center of the substrate [14]. The first item (x) and the common difference (d) are 2 mm and 1.8 mm, respectively. The distribution of S-type chips, as the name implies, is in an “S” shape, with the distance between the chips consistent with the arithmetic arrangement (x = 2 mm; d = 1.8 mm). However, unlike the arithmetic arrangement, the “S” type effectively increases the actual distance between the two chips, which can enhance the heat dissipation of the chips while improving the illumination of the LED filament. The combination type of equal intervals differs from the distribution type of equal intervals: firstly, this method blends two chips with a distance of x (x = 2 mm) at one combination; secondly, the distance between this combination and other combinations is y (y = 12 mm); finally, as the central part of the substrate has the most heat, the chips in this region are arranged equidistantly (y = 12 mm). The schematic diagram of the four chip distribution packages is shown in Figure 8.

**Figure 9:**
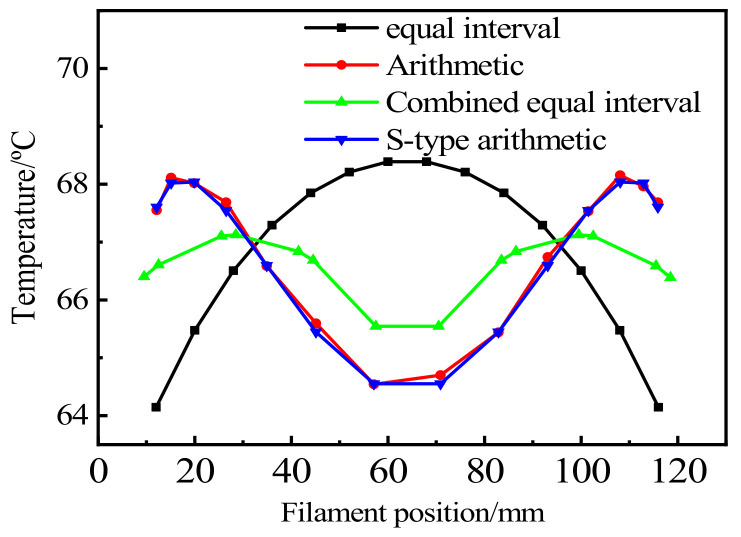
Temperature distribution of the chip.

The finite element analysis results show that the highest temperature of LED filaments distributed at equal intervals is 68.388 °C, and the lowest temperature is 60.808 °C. The maximum temperature of the LED filament of the equidistant distribution is 68.038 °C, and the minimum temperature is 62.653 °C. The maximum temperature of the S-type equal difference is 68.154 °C, and the minimum temperature is 62.611 °C. The maximum temperature of the combined equal-spaced LED filament is 67.127 °C, and the minimum temperature is 63.609 °C. It can be seen from the above results that the maximum temperature of LEDs can be arranged from high to low as: equal interval > S-type equal difference > equal difference > combined equal interval. The three types of LED chip distributions proposed attain better heat dissipation, with the combined-type having the best performance. The temperature distribution of the chip is given in Figure 9. The difference between the maximum temperature and the minimum temperature of the conventional LED filament is the largest; additionally, the heat dissipation is uneven, which contributes to most of the chips being at a higher temperature. In the arithmetic and S-type arithmetic arrangements, the temperature of the middle chip of the LED filament is lower. Compared to the other two methods, the middle chip has the lowest temperature for longer durations. Further, the temperature on both sides is also decreased, which is conducive to the diffusion of heat to both sides rather than in only one direction. The minimum temperature difference and the significant heat dissipation effect of the combination are displayed in Figure 9. This method reasonably distributes the superposition of the LED heat so that the heat of the chip at each position is small, which can play a role in protecting the chip.

### 3.4. Impact of Arrays on LED Filament Packaging

**Figure 10:**
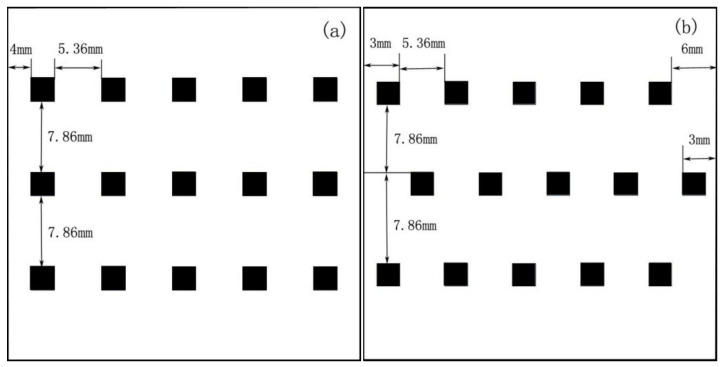
(**a**) Model diagram of a rectangular array; (**b**) model diagram of a diamond array.

Differing the LED chip array also affects heat dissipation and illuminance [29]. Most LED filaments on the market are single-row. The LED filament is converted into a square LED filament with constant surface area and volume, which is directly installed inside the LED bulb and connected to the socket. We configure the number of LED chips to 15 to facilitate research and calculation. The rectangular and diamond arrays of LED filament chips are analyzed with the same simulation conditions. The chips in the rectangular array are equally spaced from side to side and from top to bottom; the chips in regular columns are shifted to the right in the diamond array. The model diagram is shown in Figure 10.

The two common arrays and the traditional LED filaments are subjected to the finite element simulation. The simulation shows that the maximum temperature of the traditional array package is 70.063 °C, and the maximum temperatures of the rectangular and diamond arrays after the substrate conversion are 69.689 °C and 69.702 °C, respectively. The simulation results of the diamond and the rectangular arrays are similar, which matches the results of Chen et al. [30], whose study of the illuminance distribution of three typical LED array (diamond, ring, and honeycomb) light sources in the near field found that the diamond array can obtain a wide range of flatness.

## 4. Optical Analysis of LED Filament

Changing the material and structure of the substrate reduces the junction temperature of the LED filament well, while the conversion of the LED array has a negligible effect on heat dissipation. In addition, changing the spatial distribution of LED chips cannot only dissipate heat, but also address the issue of thermal superimposition in the center of the substrate. Therefore, in the design of the LED filament, high heat dissipation efficiency and uniform temperature distribution is achieved by changing the substrate material and the spacing of the LED chips. The illuminance and uniformity of the LED package also need to be further analyzed after solving the heat dissipation and temperature unevenness. This study is primarily focused on LED illumination under different chip distributions rather than the influence of different substrates on the LED light flux.

### 4.1. Illumination Analysis

**Figure 11:**
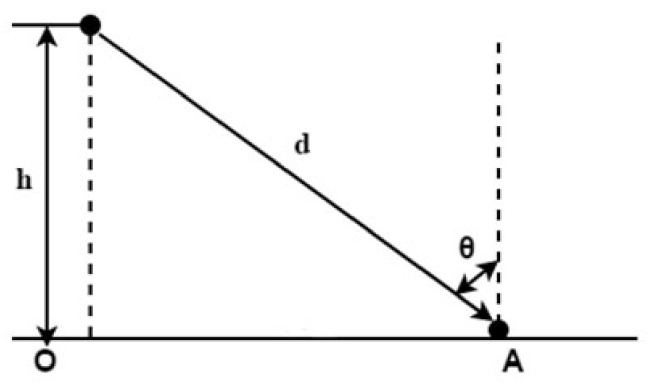
The inverse square law of distance.

A light source can be regarded as a point source when the source size is less than one-fifth the distance from the source to the calculated point. As the vertical distance between the illuminated point and the chip is 49 mm, and the chip size is only 1 × 1 × 0.25 mm^3^, the light can be counted as a point source in this experiment. Illumination calculation [31] mainly uses the inverse square law of distance. As shown in Figure 11, the horizontal illuminance generated by the point source at point A is expressed by the equation.

(2) $$E=\frac{I_{\theta }}{d^{2}}\cos\theta$$

In the formula, *E* represents the illuminance, $I_{\theta }$ is the maximum light intensity in the light intensity distribution of the LED package, d is the distance from the point light source to point A, and θ is the angle between the point light source and the vertical direction. Because the distance from the point light source to point A is difficult to measure, it can be represented by the vertical distance, h, between the point light source and the illuminated surface. Therefore, the horizontal illuminance generated by point A can also be expressed by the following formula [31]:

(3) $$E_{h}=\frac{I_{\theta }}{h^{2}}\cos^{3}\theta$$

The vertical illuminance produced at point A (from A to 0) is:

(4) $$E_{V}=\frac{I_{\theta }}{l^{2}}\sin\theta =\frac{I_{\theta }}{h^{2}}\sin\theta \cos^{2}\theta$$

The LED filament is composed of multiple point-light sources. When multiple point-light sources illuminate the work surface at the same time, the illuminance generated by all the light sources is equivalent to the sum of the illuminance generated through each light source [31]. The light intensity distribution employed in the calculation is that of an ordinary LED, and the maximum light intensity is 12.5 cd. The calculation results for the illuminance of the LED filament package are listed in Table 2.

Illumination can be subdivided into vertical illumination and horizontal illumination. The results demonstrate that the vertical illuminance levels are arranged from high to low as follows: S-type equivalence > Arithmetic > Combined equal interval > Equal interval. Further, the vertical illuminance of the arithmetic LED arrangement is estimated to be 6.7% higher than the equal interval. At the same time, the S-type equivalence arrangement increased by 9.3% compared to the equal interval; the combined equal-space interval increased by 5.0%. Therefore, the S-type equivalence attains the highest illumination. Horizontal illuminance levels are arranged from high to low as follows: Equal interval > Combined equal interval > Arithmetic > S-type arithmetic. The ratio of horizontal to vertical illuminance is mainly to ensure the lighting effect and the three-dimensional sense of lighting. Researchers can design lamps according to the horizontal and vertical illuminance of these four models, which can be more accurately applied to each aspect.

### 4.2. Uniformity Analysis

Illuminance is not the only measure of LED brightness and reliability. To meet the visual requirements of people, illumination uniformity must be taken into consideration. Illumination uniformity refers to the ratio of the minimum illuminance to the average illuminance on a given surface [32]:

(5) $$\lambda =\frac{E_{\min}}{E}$$

The uniformity of light distribution affects the lighting effect, visual experience, and uniformity of the light source. Generally speaking, a uniformity of illumination close to one indicates a light source that is less harmful to peoples’ eyes. The uniformity of the horizontal illuminance of the four spatial distribution methods of equal distance, equal difference, S-type equal difference, and combined equal distance are 0.44, 0.44, 0.47, and 0.43, respectively, and the uniformity of vertical illuminance is 0.06, 0.06, 0.32, 0.06. It is concluded that the S-type equal difference not only has the largest illuminance, but also reaches the best uniformity of vertical and horizontal illuminance.

## 5. Conclusions

A finite element thermal model of an LED filament is created in this study. The finite element steady-state analysis is utilized to analyze and compare several methods that affect the heat dissipation of the LED filament package. The LED heat dissipation and lighting capacity are calculated through the LED-related theory. Conclusions are as follow:

(1) The middle temperature of the LED filament package is high and decreases towards both sides.
(2) Among non-metallic materials, the heat dissipation capacity of the AlN substrate is the best; without considering the factor of the insulating layer, the heat dissipation capacity of the metal Cu is better among the metal materials.
(3) To solve the problem of high cost of using metal copper, a copper-aluminum-copper composite substrate is used, and its heat dissipation effect is similar to that of Cu, and the cost is not as high as that of using metal copper directly.
(4) Adding heat dissipation holes on the substrate will also increase the temperature difference, and the uniformity of temperature distribution is suboptimal.
(5) The three-chip arrangements of the arithmetic, the S-type arithmetic, and the combined equidistant distance not only solve the problem of the cumulative temperature of the LED filament and the uneven temperature distribution, but also enhance heat dissipation. Among them, the combined equidistant LED filament has the best heat dissipation performance and the lowest junction temperature.
(6) Three-chip array improves the heat dissipation and increases the vertical illuminance. The S-type arithmetic LED filament has large illumination and good uniformity, which can provide a better visual experience.

## Figures and Tables

**Table 1:** Thermal conductivity of different substrate materials.

Substrate Material	Al_2_O_3_	AlSiC	Si	AlN	Al	Cu
Thermal conductivity (W·m^−1^·K^−1^)	20	160	149	200	240	401

**Table 2:** Illuminance of LED filaments in four modes of package.

Distribution Method	Vertical Illuminance/Lx	Horizontal Illumination/Lx
Equal interval	217.93	472.93
Arithmetic	232.43	413.65
S-type arithmetic	238.02	412.12
Combined equal interval	228.70	428.60

## Data Availability

Not applicable.
